# Intelligent Fault Diagnosis of Rotary Machinery by Convolutional Neural Network with Automatic Hyper-Parameters Tuning Using Bayesian Optimization

**DOI:** 10.3390/s21072411

**Published:** 2021-03-31

**Authors:** Davor Kolar, Dragutin Lisjak, Michał Pająk, Mihael Gudlin

**Affiliations:** 1 Faculty of Mechanical Engineering and Naval Architecture, University of Zagreb, Ivana Lučića Street 5, 10002 Zagreb, Croatia; dragutin.lisjak@fsb.hr (D.L.); mihael.gudlin@fsb.hr (M.G.); 2 Faculty of Mechanical Engineering, University of Technology and Humanities in Radom, Stasieckiego Street 54, 26-600 Radom, Poland; m.pajak@uthrad.pl

**Keywords:** rotary machinery, fault diagnosis, convolutional neural network, classification, hyper-parameters tuning, bayesian optimization

## Abstract

Intelligent fault diagnosis can be related to applications of machine learning theories to machine fault diagnosis. Although there is a large number of successful examples, there is a gap in the optimization of the hyper-parameters of the machine learning model, which ultimately has a major impact on the performance of the model. Machine learning experts are required to configure a set of hyper-parameter values manually. This work presents a convolutional neural network based data-driven intelligent fault diagnosis technique for rotary machinery which uses model with optimized hyper-parameters and network structure. The proposed technique input raw three axes accelerometer signal as high definition 1-D data into deep learning layers with optimized hyper-parameters. Input is consisted of wide 12,800 × 1 × 3 vibration signal matrix. Model learning phase includes Bayesian optimization that optimizes hyper-parameters of the convolutional neural network. Finally, by using a Convolutional Neural Network (CNN) model with optimized hyper-parameters, classification in one of the 8 different machine states and 2 rotational speeds can be performed. This study accomplished the effective classification of different rotary machinery states in different rotational speeds using optimized convolutional artificial neural network for classification of raw three axis accelerometer signal input. Overall classification accuracy of 99.94% on evaluation set is obtained with the CNN model based on 19 layers. Additionally, more data are collected on the same machine with altered bearings to test the model for overfitting. Result of classification accuracy of 100% on second evaluation set has been achieved, proving the potential of using the proposed technique.

## 1. Introduction

Fault diagnosis plays an essential role in relating monitoring data with the health states of the machinery [1], that is known to be a key issue in machine health monitoring process. The relation between data and the machine state can be done using experience engineer that can discover differences in machine monitoring data that can be related to machine health. However, the development of sensor industry, communication protocols and Industrial Internet of Things leads to a lower price and greater availability of sensors and data acquisition and processing systems, consequently leading to the greater ability to extract knowledge from these available data. With the increase in the amount of condition data collected, it is possible to create data-driven models, that is, models that describe the system in operation and can provide accurate diagnosis result based solely on the previously collected data. They are becoming suitable even for the complex systems and are receiving more and more attention from the researchers and engineers. For the particular matter of fault diagnosis, the procedure is expected to be intelligent enough to automatically detect and recognize the health states of the machines [2,3].

Intelligent fault diagnosis (IFD) refers to applications of machine learning theories, such as artificial neural networks (ANN), support vector machine (SVM), and deep neural networks (DNN), to machine fault diagnosis [4]. In last few years, researchers are beginning to exploit the potentials of deep learning and convolutional neural networks in fault identification and diagnostics, with the aim of reducing or eliminating the shortcomings of shallow ANN architectures [5], which is a step forward to intelligent fault diagnosis. Deep learning stands for class of machine learning techniques specific by its many layers of information processing stages in deep architectures that are exploited for pattern classification and other tasks [6]. However, applying the deep learning models raises a new challenge in the area of model hyper-parameter tuning [7].

Deep learning is considered to be a black box approach in which the researcher does not have much scope for hand tuning the parameters as the layers are hidden and there are many hyper-parameters related to network structure and training algorithms as well [8]. Selection of appropriate hyper-parameters values is important since they directly control the behavior of training algorithms and have significant impact on the model performance [9]. Bayesian optimization is a very effective technique for solving this kind of optimization problem [10] and outperforms other global optimization algorithms [11]. In this paper, we attempt to automatically optimize hyper-parameters and architecture of multi-channels deep convolutional neural network by using Bayesian optimization [12].

The contributions of this paper are summarized as follows:

(1.) A modular Multi-Channels Deep Convolutional Neural Network (MC-DCNN) architecture for rotary machinery state classification is developed. It is used to learn features of the raw accelerometer data thus eliminates necessity expert knowledge in vibration signal preprocessing. Network architecture is modular and hyper-parameters dependable, so it can be automatically and optimally adjusted to input data in the learning process using hyper-parameters tuning procedure.
(2.) Convolutional neural network model generally operates as black-box and requires hyper-parameters for machine learning process, hence relevant hyper-parameters for optimization process as well as optimization procedure using Bayesian optimization is proposed.
(3.) Hyper-parameters optimization using the developed procedure is conducted and results are presented.

The main motivation of this study is to find optimal Convolutional Neural Network (CNN) architecture and hyper-parameters values that can yield the best performance in intelligent fault diagnosis of rotary machinery without manually adjusting network structure any hyper-parameters.

This paper is organized as follows—in the next subchapter, related work regarding the application of CNN in fault diagnosis of rotary machinery and hyper-parameters optimization is presented. Section 2 and Section 3 reveals CNN architecture and Bayesian optimization, respectively. In Section 4, experimental setup and collected data are explained. Section 5 explains the results of the research. Finally, conclusions are drawn in Section 6.

### Related Work

Convolutional neural networks (CNN) are biologically inspired deep feed-forward artificial neural network (ANN) that present a simple model for the mammalian visual cortex. CNNs are proposed by LeCun et al. [13] and now widely used and virtually have become the standard in many object recognition systems in an image or video. As supervised deep learning technique, CNN accomplished superior results in image identification, speech recognition and target tracking [14]. In general, convolutional neural networks consist of convolutional layers, pooling layers and full-connected layers [15] and the way it works is explained in detail in [16].

**Figure 1:**
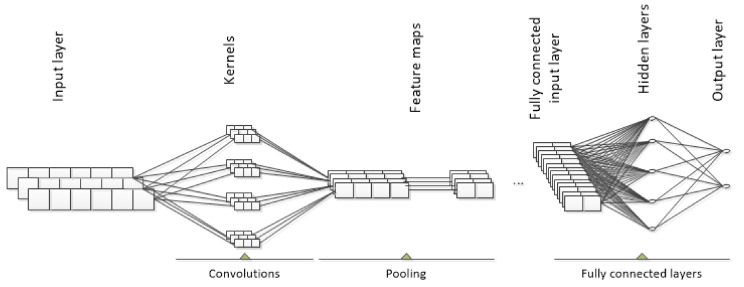
Generic structure of multi-channels 1D Convolutional Neural Network (CNN).

Currently, many papers can be found in the field of predictive maintenance that are dealing with the application of convolutional neural networks for the intelligent fault diagnosis. In the terms of the architectures of CNN, they can be classified into the 1-dimensional (1D) fault diagnosis models and 2-dimensional (2D) fault diagnosis models. CNNs were originally developed for image and video classification, thus 2D CNN inputs are images in two dimensions. For intelligent fault diagnosis, researchers adopted CNN so it can handle machine monitoring data that is most commonly collected as 1D signal. Researchers using 2D architecture additionally explored either signal processing methods [17,18,19,20], manually constructed signal matrix [21,22] to enable CNN or used health state image like infrared thermal image [23] or grey scale image [24]. The use of such techniques has enabled the applicability of the CNN model in intelligent fault diagnosis, as is the case in image classification. Compared to 2D CNN models, 1D fault diagnosis models work with raw sensor data that are described as 1D time series and can avoid preprocessing with the aforementioned techniques. 1D CNN techniques for induction motors [25,26], pumps [27] and rolling element bearings [28,29] are developed. Further on, the authors of [30] presented multi-channels 1D CNN (MC-DCNN) for human activity classification that is modified by [31] for 3 axis vibration data input with input size of 6400 × 1 × 3. This type of CNN is visualized in Figure 1. Input layer consist of three channels input and the length of each input is 6400. Convolutional layers compute the output of the neurons, while pooling pass over sections of vibrational signal and pool them into the highest value in the section. Full-connected layer classify data in one of the previously defined classes. Defining the model architecture can be difficult since there are multiple architecture options available and researcher does not know optimal structure or hyper-parameters values. In this research, authors perform additional modifications in input size as well as in MC-DCNN architecture previously presented in [31] to enable intelligent fault diagnosis and optimization of both network structure and hyper-parameters.

Machine learning algorithm transforms a problem that needs to be solved into an optimization problem that uses different optimization methods. Optimization function is a compound of multiple hyper-parameters that are set before the learning process and influences how to algorithm fits the model to the data. Unlike internal model parameters, such as weights in neural networks, hyper-parameters cannot be learned from the data during training process. The influence of hyper-parameters on training accuracy and speed suggest that they must be configured before the training process begins. The process that yields the hyper-parameters for specific training data is called hyper-parameter optimization and it can be defined as an optimization problem where the objective function is an unknown or black-box function. Until now, there has been no standard method for optimal hyper-parameter selection, because there is no clear relationship between model performance and hyper-parameters [7,32]. To overcome this drawback, it is possible to train multiple models with different hyper-parameters values and find the best combination of it by comparison. Having in mind that hyper-parameters values have a significant impact on classification accuracy of the model, a way to optimize hyper-parameters becomes a hot issue in a machine learning process. Some research has been done on optimizing hyper-parameters [33,34,35], mostly by using standard CIFAR-10 dataset. Hyper-parameter tuning can be done manually or automatically. While manual search requires and depends on expert knowledge and practical experience, automatic hyper-parameter search completely remove human from machine learning process. Automation of the hyper-parameters selection reveals its clear advantages in cases of a larger number of hyper-parameters, where even experts are not capable of handling high dimensional data and relationship between them. The state-of-the-art algorithms for hyper-parameters optimization can be classified into two categories—search algorithms and trial schedulers. Mainly, search algorithms are applied for sampling while trial schedulers deal with the early stopping methods for model evaluation [36].

Grid search algorithm trains a model with each combination of possible values of hyper-parameters and outputs the hyper-parameters values that achieve the best performance during training. Although this method works automatically and in theory can find the global optima of objective function, it is not effective. Random search as another type of search algorithm tries to reduce deficiencies of grid search in term of expensive cost. It reduces search to a subset of hyper-parameters which have the most influence on results. Albeit it is more efficient with greater number of hyper-parameters, some research [33] shows that it can be unreliable for training complex models. Additionally, grid and random search are completely unaware of previous evaluations. In contrast to Bayesian grid and random search, Bayesian optimization stores and includes past results in evaluation of the hyper-parameters values. Authors in [8,9] investigate automatic tuning of hyper-parameters on different machine learning architectures including CNN and define Bayesian optimization as usable method for hyper-parameters tuning. This research altogether suggests that the challenge of automatic hyper-parameters tuning is still present in machine learning. In this paper, the authors present the results of research into hyper-parameters tuning of the CNN architecture for fault diagnosis of rotary machines using Bayesian optimization.

## 2. CNN Hyper-Parameters

Hyper-parameters can be defined as a group of parameters that are used in the machine learning process. As noted in the introduction, hyper-parameters differ from the parameters of the internal machine learning algorithm in that they cannot be learned from data during the learning process. Hyper-parameters of the convolutional neural network can be divided into two types:

1. Network structure definition hyper-parameters such as:
  - Kernel size—size of the filter
  - Number of kernels—number of filters
  - Stride—the rate at the filter jumps over the input image
  - Padding—adding borders of zeros to input images
  - Number of hidden layers—layers between input and output
  - Activation functions— function that allows model to learn nonlinear boundaries
2. Network training process hyper-parameters such as:
  - Learning rate—regulates the update of the weights after each batch
  - Momentum—regulates the influence of previous weights update on the current update
  - Number of epochs —number of iterations of learning
  - Batch size—the number of samples shown to the network before weights update

Hyper-parameters values can have a major impact on the model. To the authors’ knowledge, there is no set of best hyper-parameters that fits for all models, yet the set of hyper-parameters should be in the right combination of values having minimum loss function or maximizing the performance or accuracy of the model. Thus, hyper-parameters optimization can be formulated as an optimization problem and solved using optimization algorithms. In this work, the authors considered the learning rate, momentum, kernel size and the number of kernels from the list of the standard hyper-parameters of the CNN to be optimized. Architecture of the CNN can be modified using additional hyper-parameters, as is explained in Section 5.1.

## 3. Bayesian Optimization

In general, there are two classes of hyper-parameter optimization methods, that is, manual and automatic search methods. Manual hyper-parameters optimization is a hard procedure to reproduce since it is based-on many attempts of trial and error. Grid search is not scalable for higher dimensions. Random search acts like the greedy approach, settling for local optima and thus not reaching to global optima. Other evolutionary optimization methods require a greater number of training cycles and can be noisy. As stated earlier, Bayesian optimization can overcome all these constraints by efficiently finding the global optima of the black box function of the neural network and it is derived from Bayes theorem. Bayesian optimization is a method for solving functions which are computationally expensive to find the extrema [37].

The key elements in the optimization process are:

- A Gaussian process model of f(x).
- A Bayesian update procedure for modifying the Gaussian process model at each new evaluation of f(x).
- An acquisition function a(x) based on the Gaussian process model of *f* which is maximized to determine the next point *x* for evaluation.

By using such a mechanism it can be concluded where the function obtains the optimal value thus reducing loss and maximizing the model’s accuracy. As stated, in this paper the optimization goal is to find the minimum value of the loss at the sampling point for an unknown function *f*:

(1) $$\begin{array}{c} \begin{array}{c} x_{opt}=\arg\;\underset{x\in D}{min}\;f\left(x\right), \end{array} \end{array}$$

where *D* denotes the search space of *x*.

The underlying probabilistic model for objective function *f* is a Gaussian process prior with added Gaussian noise in the observations that is explained in detail in [34]. Gaussian process is a generalization of Gaussian probability distribution, where any finite sub-collection of random variables has a multivariate Gaussian distribution [38]. Gaussian process works in a way that expects outputs similar to inputs, and thus assumes a statistical model of the function.

(2) $$\begin{array}{c} \begin{array}{c} P(M|E)\infty P(E|M)P(M). \end{array} \end{array}$$

Equation (2) reflects the idea of Bayesian optimization. Looking into sample data *E*, posterior probability P(M|E) of a model *M* is proportional to the probability P(E|M) of observing *E* given model *M* multiplied by the prior probability of P(M). It can be concluded that Bayes optimization optimizes unknown function by combining the prior distribution based on Gaussian process of the function f(x) with the current sample information to obtain the posterior of the function. In the next step, the posterior information is used to find where the function f(x) is minimized through criterion value. The criterion is represented by a utility or acquisition function *a*. The function *a* is used to define the next sample point in order to maximize the expected utility. There are few commonly used acquisition functions. In this paper, expected improvement (*EI*) function is used, as it evaluates the expected amount of improvement in the objective function, ignoring values that cause an increase in the objective. Function *EI* calculates the expectation of the degree of improvement that a point can achieve when exploring the vicinity of the current optimum value [39]. If $x_{best}$ is the location of the lowest posterior mean and $\mu_{Q}\left(x_{best}\right)$ is the lowest value of the posterior mean than the expected improvement is

(3) $$\begin{array}{c} \begin{array}{c} EI\left(x,Q\right)=E_{Q}\;\left[max\left(0,\mu_{Q}\;\left(x_{best}\;\right)-f\left(x\right)\right)\right] \end{array} \end{array}$$

In other words, if the improvement of the function value is less than the expected value after the algorithm is executed, then the current optimal value point may be the local optimal solution, and the algorithm will find the optimum value point in other positions of the domain. Searching the sampling area includes both exploration (sampling from the areas of high uncertainty) and exploitation (sampling from that with high values) [40], which help in reducing the number of samplings. Finally, the performance will be improved even when the function has multiple local maxima. In addition to the sample information, Bayesian optimization depends on the prior distribution of the function *f*, which is a required part in the statistical inference of the posterior distribution of the function *f*.

The main steps in the optimization are as follows:

1. For current iteration *t*
2. Evaluate $y_{i}=f\left(x_{i}\right)$ for defined number of points $x_{i}$ taken at random within the variable bounds.
3. Update the Gaussian process model of f(x) to obtain a posterior distribution over functions Q(f|xi,yifori=1,…,t).
4. Find the new point *x* that maximizes acquisition function a(x).

The algorithm stops after either reaching time or number of iterations limit.

In this paper, tuning the hyper-parameters and CNN architecture is achieved using Bayesian optimization (Bayes Opt) algorithm presented in [12].

## 4. Experimental Setup and Analysis

**Figure 2:**
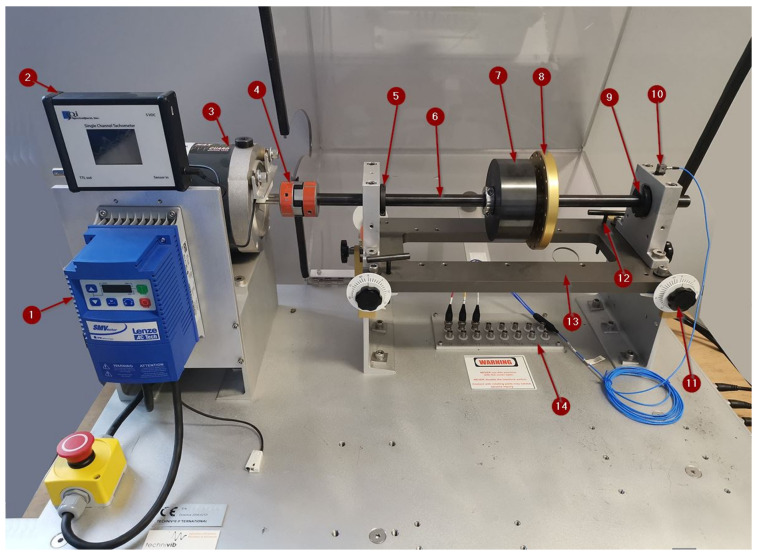
Fault simulator (1. Frequency drive Lenze SMVector; 2. Tachometer display, 3. Motor, 4. Clutch, 5. Front-end bearing, 6. Main shaft, 7. Load, 8. Main rotor, 9. Back-end bearing, 10. Three-axis IEPE accelerometer, 11. Horizontal axis alignment screw, 12. Vertical axis alignment screw, 13. Base, 14. BNC connectors).

The experimental setup used for machine condition simulation and data acquisition consist of rotary machinery fault simulator and data acquisition system. A SpectraQuest variable speed Machinery Fault Simulator (MFS) was used as experimental setup data generator for both normal operation and faulty condition data. The system (illustrated in Figure 2) consist of a 0.75 kW variable speed motor driving a shaft-rotor component via coupling supported with two sets of *ER12K* ball bearings. The basic configuration consisted of main rotor positioned centrally on main shaft. As different modules representing different rotary machinery states can be mounted on a device, it can be used for emulating different real world rotary machinery fault scenarios. The experimental configuration of MFS is outfitted with three-axis accelerometer and a tachometer, that are connected to a National Instruments DAQ System. In addition to the above-mentioned failure simulator modules, an inertial disk weighing 5 kg was additionally used with the aim of increasing the basic load of the entire system.

Although there are several other types of sensors that can serve as data sources for condition monitoring, accelerometers monitoring vibrations have been selected and used in this study. They have been chosen for the fact that vibration monitoring is considered the most powerful predictive maintenance technique [41]. Three-axis PCB Piezotronics 356B21 IEPE type accelerometer is mounted on the bearing housing on the shaft side opposite of the motor position. The sampling frequency is set to 51.2 kHz, while revolving speed during the experiment is set to 1000 r/min and 1500 r/min, respectively. Vibration signals in three directions are acquired when the system operates under 16 different conditions. Each particular condition is combination of rotational speed and state of the machine. Non-normal states of the machine are simulated by coupling additional modules or faulty parts to the machine. Each acquired sample of 12,800 data points is stored and labeled as data set representing particular condition, that is, combination of rotational speed and machine state. Simulated fault conditions with descriptions and labels are listed in Table 1. As it can be seen in the table, situations with both normal behavior of the machine and faulty conditions are observed. Condition labels are used in machine learning process as class indicators. For the purpose of the experiment, main shaft is loaded with main rotor and additional load, as it can be seen in Figure 2. If the system operates in normal behavior, that is, there are no faults, our model should predict normal machine state. For the purpose of simulating rotor faults, additional faulty rotors are mounted. Debalanced rotor is simulated by adding extra weight of 20 g in main rotor in previously defined position. Different bearing fault are simulated using bearings with inner or outer race faults, as well as bearing running with ball bearing fault. Additionally, bearing with faults on both inner and outer races combined with ball bearing fault is used. Machine bearings were provided by MFS manufacturer and seeded with faults using electro-discharge machining (EDM). 1500 samples of each condition is collected. In total, 24,000 data sets have been collected to train, optimize hyper-parameters and test the convolutional neural network data-driven model for failure classification.

Signals are collected and divided for optimization and evaluation data sets separately for each condition by using stratified sampling before being used in optimization and evaluation procedure. Samples for the final evaluation of the model were not used in the hyper-parameter optimization process.

**Figure 3:**
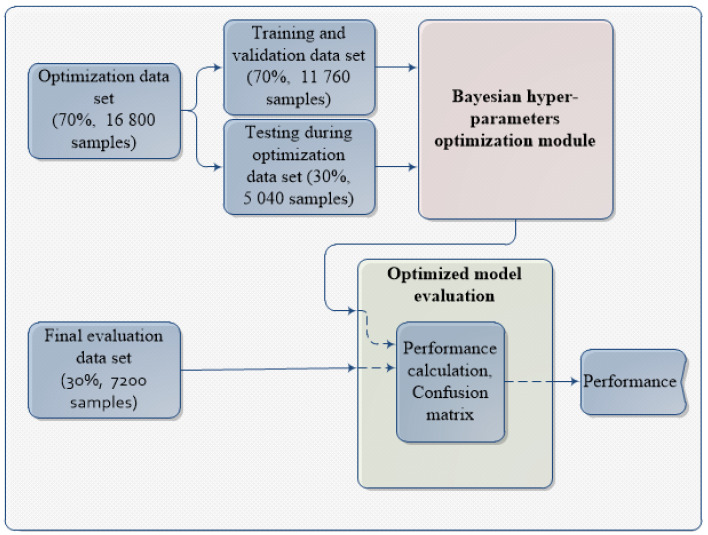
Optimization and evaluation process outline.

The whole optimization and evaluation process is outlined in Figure 3.

From all the samples, 70% of the data are used in the optimization process (training, validation and testing during optimization) while rest of 30% is used for final testing of the model and 30% of the training data are used for model testing during optimization procedure, while 10 % of the training data are used for validation during training procedure.

### Hyper-Parameters Optimization

In this chapter, the optimization process for fault diagnosis model is explained in detail. As stated in introduction, the main goal of this study is to define algorithm or module that can automatically create optimal CNN architecture and hyper-parameters values, that is, CNN based model that can yield the best performance in intelligent fault diagnosis of rotary machinery without manually adjusting network structure and hyper-parameters. As a result of literature research, Bayesian optimization (Bayes Opt) [12] has been chosen as the optimization technique.

**Figure 4:**
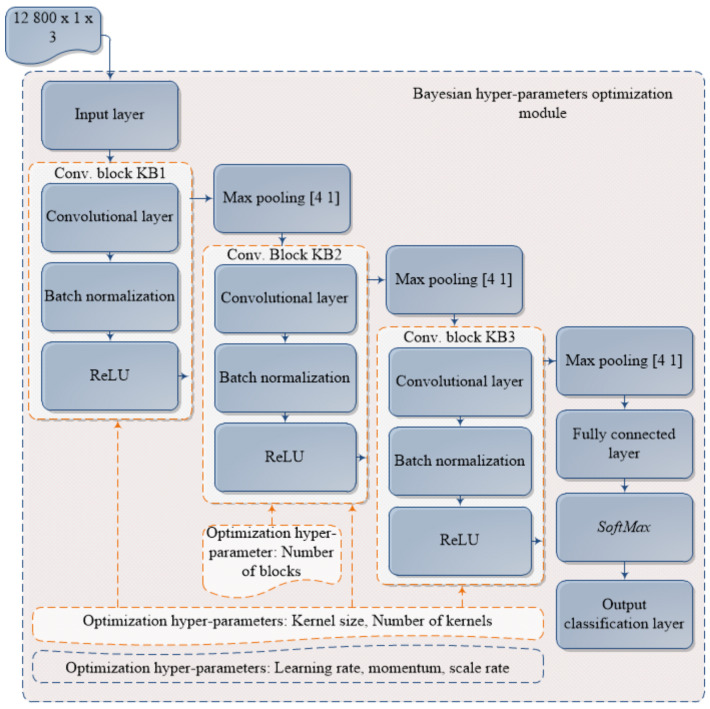
Model outline for hyper-parameters optimization of fault diagnosis CNN model using Bayesian optimization.

Figure 4 presents a proposed model of Bayesian hyper-parameters optimization of CNN for fault diagnosis. The objective of the proposed model is to find the optimal structure and hyper-parameters of CNN for intelligent fault diagnosis. The convolutional neural network used in this research is modified multi-channels 1D CNN, explained in the authors’ earlier work [31]. For the purpose of this research, the network is modified in a way it can simultaneously input raw vibration signal with size of 12,800 in each of three input channels. CNN architecture is set up with three convolutional blocks and changing the architecture layout by adding additional KB2 level of blocks by hyper-parameter Numberofblocks value is possible. By altering Numberofblocks hyper-parameter value in defined range, optimization algorithm can change network architecture by adding or subtracting KB2 type blocks. Additionally, network structure hyper-parameters Kernelsize and Numberofkernels can be tuned for all convolutional blocks. Finally, network training hyper-parameters Learningrate, momentum and scalerate can also be tuned within set boundaries.

To achieve above mentioned objective, algorithm workflow presented in Algorithm 1 is used. Algorithm inputs are CNN base architecture pointed out in Figure 4, CNN hyper-parameters search space, data needed for optimization pointed out in Figure 3 as well as the algorithm hyper-parameters *Number of iterations*, *Time limit* and *Acquisition function*, respectively.
**Algorithm 1** Algorithm workflow for automatic hyper-parameters and network structure optimization.Inputs: CNN base architecture, Hyper-parameters search space, Training Data, Testing during Optimization Data, Validation Data, Number of iterations, Time limit, Acquisition function Outputs: Hyper-parameters values $\begin{array}{c} 1:\;\mathrm{procedure}\;\mathrm{Optimization}\;\\ 2:\;\;\;\;\mathrm{Assume}\;\mathrm{Gaussian}\;\mathrm{Process}\;\mathrm{prior}\;\mathrm{on}\;\mathrm{the}\;\mathrm{objective}\;\mathrm{function}\;f\;\;\\ 3:\;\;\;\;\mathrm{Find}\;\mathrm{and}\;\mathrm{evaluate}\;\mathrm{the}\;\mathrm{objective}\;f\;\;\mathrm{at}\;x_{i}\;\mathrm{number}\;\mathrm{of}\;\mathrm{points}\\ 4:\;\;\;\;\mathrm{while}\;j\in i+1\;...,N\;\mathrm{do}\;\;\;\;\;\;\;\;\;\;\;\;\;\;\;\;\;\;\;\;\;\;\;\;\;\;\;\;\;\;\;\;\;\;\;\;\;\;\;\;\;\;\;\;\;\;\;\;\;\;\;\;\;\;\;\;\;\;\;\to \;\mathrm{search}\;\mathrm{space}\;\mathrm{exploration}\\ 5:\;\;\;\;\;\;\;\;\;\mathrm{Update}\;\mathrm{the}\;\mathrm{posterior}\;\mathrm{distribution}\;\mathrm{on}\;f\;\mathrm{using}\;\mathrm{the}\;\mathrm{prior}\\ 6:\;\;\;\;\;\;\;\;\;\mathrm{Choose}\;\mathrm{the}\;\mathrm{next}\;\mathrm{sample}\;x_{j}\;\mathrm{that}\;\mathrm{maximizes}\;\mathrm{the}\;\mathrm{acquistion}\;\mathrm{function}\;\mathrm{value}\\ 7:\;\;\;\;\;\;\;\;\;\mathrm{Evaluate}\;y_{j}=f\left(x_{j}\right)\\ 8:\;\;\;\;\mathrm{return}\;x_{j}\;\;\;\;\;\;\;\;\;\;\;\;\;\;\;\;\;\;\;\;\;\;\;\;\;\;\;\;\;\;\;\;\;\;\;\;\;\;\;\;\;\;\;\;\;\;\;\;\;\;\;\;\;\;\;\;\;\;\;\;\;\;\;\;\;\;\;\;\;\;\;\;\;\;\;\;\;\;\;\to \;\mathrm{return}\;a\;\mathrm{point}\;\mathrm{with}\;\mathrm{best}\;\mathrm{hyper}-\mathrm{parameters}\;\mathrm{values} \end{array}$

As previously stated, Bayesian optimization procedure expects search space definition, that is, boundaries for hyper-parameters tuning. Based on this, optimization variables and their ranges are created, which are visible in Table 2. Subset of hyper-parameters included in this research, as well as the initial boundaries were selected based on expert experience and evaluation. As can be seen, if only integer hyper-parameters and their values are taken into account, the total number of possible combinations is 18,000, while with the inclusion of momentum and learning rate as continuous variables there are practically countless number of hyper-parameters combinations.

The optimization variables used as hyper-parameter values when performing Bayesian optimization are shown in Table 3. The use of variables to adjust the number of kernels and the size of the kernels at the convolutional blocks levels in combination with the variable of the scaling factor ultimately reduces the total number of optimization variables, that is, reduces the calculation time of the Bayesian optimization. A series of preliminary experiments confirmed the concept initially presented in [42], according to which a better model response is possible in the case of a smaller number of larger cores within the first of the convolution blocks. Therefore, in addition to the standard hyper-parameters of the convolutional neural network model itself, a scaling factor variable was added within the optimization variables of the Bayesian optimization. This optimization variable allowed to scale the hyper-parameters of convolutional blocks and create relationships in sizes between core sizes and the number of cores in individual layers.

Additionally, Bayesian optimization requires a definition of the input hyper-parameters of the optimization algorithm, which are selected as stated in Table 4.

## 5. Results

To demonstrate the proposed technique, this section provides results of the conduced research, optimal values of the hyper-parameters of the model and the classification performance obtained using tuned CNN model. The optimal optimization variable values and hyper-parameters obtained using Bayesian optimization are given in Table 5 and Table 6. The optimization procedure last in total 14 h and 52 min using GeForce RTX 2070 GPU hardware.

It can be seen that both learning rate and momentum of learning process are closer to the lower boundary of search space, as well as the vDepth variable. Moreover, vNumberOfKernels has been tuned to the lower bound. In addition, variable vScaleRate that is used for KB1 and KB2 structure definition is tuned to upper boundary. Variable vKS is closer to the upper search space boundary with value of 45.

**Figure 5:**
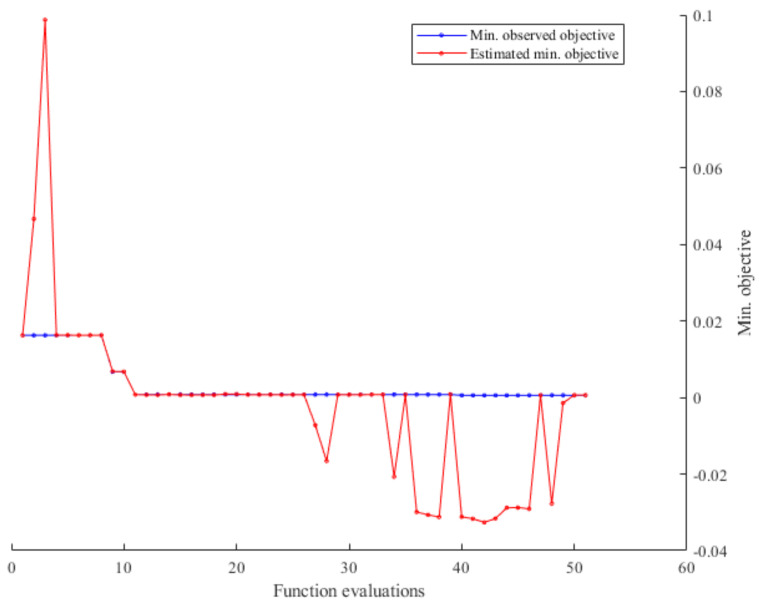
Min. objective through function evaluations.

Training of convolutional neural network is computationally expensive and time-consuming and using of GPU hardware is highly advisable. Using Bayesian optimization, acquisition function enables narrowing down the search space thus expensive function is performed in a narrowed region of values. The function evaluation that can explain achievement of minimum objective is illustrated in Figure 5. In every iteration of Bayesian optimization sample points generated by acquisition function are evaluated by objective function (Gaussian process model). As the observations accumulate, the posterior distribution is updated continuously on the basis of the new posterior, that is, the point where the acquisition function is maximized is found and added to the training data set.

**Figure 6:**
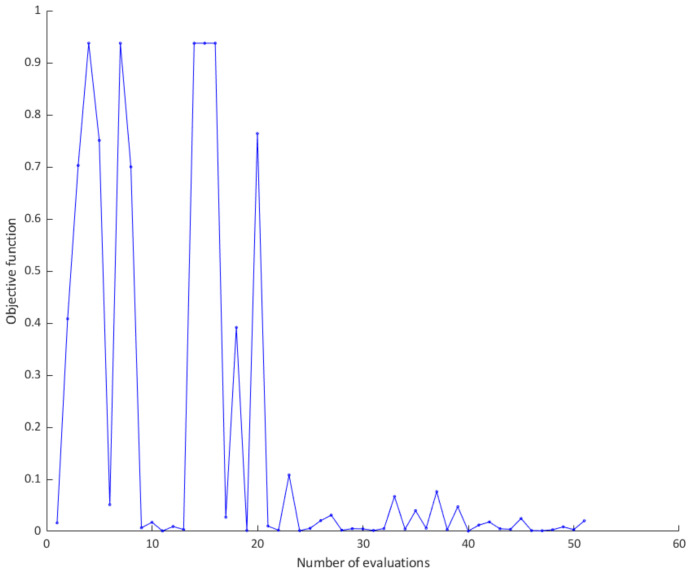
Objective function trend.

Further on, the objective function trend throughout evaluations can be seen in Figure 6. Both Figure 5 and Figure 6 shows that hyper-parameters that are optimized throughout the layers and on a network structure level ends in global optimization, thereby improving the performance of the models. It could be seen that some of the hyper-parameters combination yield in only 6.25% of model accuracy. Furthermore, it can be observed that the results vary significantly up to 21st iterations, after which the Bayesian optimization manages to determine the hyper-parameters that ensure the classification accuracy greater than 90%.

**Figure 7:**
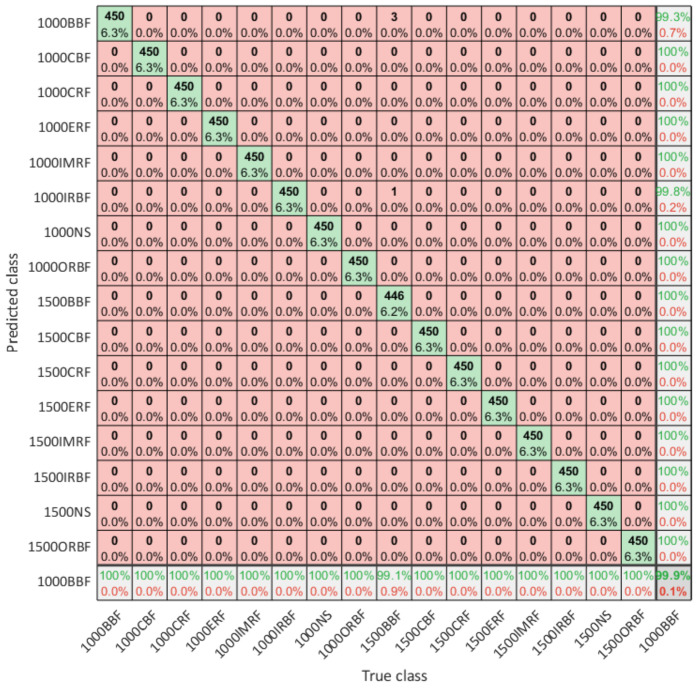
The confusion matrix for 16 predicted classes across the true class with 7200 evaluation samples.

In Figure 7, confusion matrix for classification of 7200 test samples raw accelerometer data by using optimized model is illustrated. It can be seen that optimized model incorrectly classified only 4 samples of ball bearing fault, while all other samples were classified according to true classes. In comparison with the study [31], it should be noted that the network structure declared as CNN_24-48 that yield the best results in previous study with limited number of classes but on the same experimental setup was also trained on this set of data and without any further optimization showed a classification accuracy of 81.6%. This explains the importance of using hyper-parameters and structure optimization of CNN model in case of intelligent fault diagnosis.

The optimal structure of the CNN model as a result of hyper-parameters and network structure optimization is presented in Table 7. All convolutional layers use stride equals 1 and padding is calculated so that the output has the same size as the input. In total, there are 2,461,084 learnable parameters of the model. Finally, a test data set is used with an optimized model to declare confusion matrix and calculate classification accuracy. The optimal model achieved an equal classification accuracy of 99.94% on the evaluation data set.

### 5.1. Additional Evaluation Set Results

**Figure 8:**
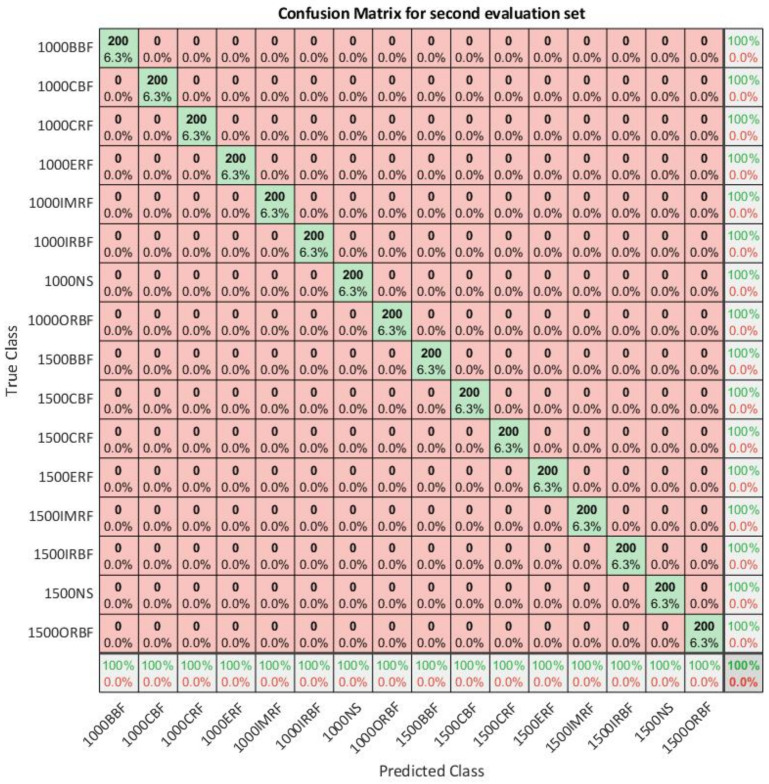
The confusion matrix for 16 predicted classes across the true class with second evaluation set.

High classification accuracy on the evaluation data can be achieved due to fact that the deep learning model can overfit the training data. As for the first evaluation data set all data were collected on the same platform, authors collected another set of evaluation data with 200 samples for each class. Experimental setup on MFS is altered in a way that another set of both faulty and non-faulty bearings were used during the data acquisition process. The results of the classification of the additional evaluation set can be seen in Figure 8. Optimized model is evaluated on the second evaluation set and an overall classification accuracy of 100% is achieved. The second evaluation of the optimized model can confirm that the model performs well on the new data, that is, data collected from the altered platform. However, it must be said that both training and evaluation data set acquisition has been done on laboratory stand in laboratory conditions and additional testing in both laboratory conditions using simulated noise and real industrial conditions are preferable.

## 6. Conclusions and Future Work

In this paper, we propose a technique for intelligent fault diagnosis of rotary machinery that uses Bayesian optimization in the optimization of hyper-parameters and the structure of a convolutional neural network. For this purpose, we presented where and how other researchers used convolutional neural networks for machinery intelligent fault diagnostics. Further on, hyper-parameters optimization possibilities are described and strengths and weaknesses of Bayesian optimization are outlined. The process for the optimization of hyper-parameters and structure of convolutional neural network model for fault diagnosis is presented. Bayesian optimization combined a prior distribution of a function with sample information (evidence) to obtain posterior of the function. Later on, the posterior information was used to find where the function was maximized according to a criterion. The results show that there is great potential in using the Bayesian optimization technique for both CNN hyper-parameters and structure optimization since it can generate optimized hyper-parameters efficiently in times when the objective function is time and computationally expensive, as is the case with convolutional neural networks. Optimal hyper-parameters values and the structure of CNN model for intelligent fault diagnosis of rotary machinery ensured 99.94% accuracy on the evaluation data set. In addition, second evaluation data set with 3200 samples is prepared using another set of both undamaged and faulty bearings on the same machine. Using tuned CNN model, classification accuracy of 100% is achieved, proving that a model trained with the proposed technique performs well on the new data set. Future work will be based on experimenting with industrial (noisy) data, data fusion from multiple sensors and genetic algorithms as optimization technique. Hence, running on multiple GPU can also be taken into consideration. In the foreseeable future, with the running of multiple Bayesian optimizations and comparison of results, it will be possible to determine the influence of hyper-parameters on the results, that is, to define the set of the most important hyper-parameters.

## Figures and Tables

**Table 1:** Simulated fault conditions and labels.

No	Machine State	Description	RPM	Condition Label
1	Normal state	Machine is running without simulated fault.	1000	1000 NS
2			1500	1500 NS
3	Debalanced rotor	Machine is running with simulated fault of imbalance on main shaft. Weight of 40 g is added to the main rotor.	1000	1000 IMRF
4			1500	1500 IMRF
5	Eccentric rotor	Fault is simulated by replacing the main rotor with rotor that have asymmetrically located central hole.	1000	1000 ERF
6			1500	1500 ERF
7	Cocked rotor	Fault is simulated by replacing the main rotor with cocked rotor (0.5 degree off-axis).	1000	1000 CRF
8			1500	1500 CRF
9	Outer race bearing fault	Machine is running with bearing outer race fault.	1000	1000 ORBF
10			1500	1500 ORBF
11	Inner race bearing fault	Machine is running with bearing inner race fault.	1000	1000 IRBF
12			1500	1500 IRBF
13	Ball bearing fault	Machine is running with bearing ball fault.	1000	1000 BBF
14			1500	1500 BBF
15	Combined bearing fault	Machine is running with both bearing races faults combined with ball fault.	1000	1000 CBF
16			1500	1500 CBF

**Table 2:** Ranges of optimization variables for Bayesian optimization.

Optimization Variable	Range	Data Type
vLearnRate (vLR)	0.001–0.01	Decimal, logaritmic
vMomentum (vM)	0.80–0.95	Decimal, logaritmic
vKernelSize (vKS)	4–64	Integer
vNumberOfKernels (vNK)	4–24	Integer
vDepth (vD)	0–5	Integer
vScaleRate (vSR)	1–3	Integer

**Table 3:** Hyper-parameter calculation using optimization variables.

Hyper-Parameter Level	Hyper-Parameter	Optimization Variable or Calculation
Network training	Learning rate	vLR
Network training	Momentum	vM
KB1 structure	Kernel size	vKS·vSR
	Number of kernels	vNK
KB2 structure	Kernel size	vKS/vSR
	Number of kernels	vNK·vSR
	Number of blocks	vD
KB3 structure	Number of kernels	vNK

**Table 4:** Bayesian optimization hyper-parameters.

Hyper-Parameter	Value
Acquisition function	Expected improvement
Exploration ratio	0.5
Number of seed points	10
Maximum number of evaluations	50
Time limit in hours	100

**Table 5:** Optimal optimization variable values and accuracy.

vLR	vM	vKS	vNK	vD	vSR	Classification Accuracy [%]
0.003245109	0.827834971	45	4	2	3	99.94

**Table 6:** Optimal hyper-parameters values of CNN.

Hyper-Parameter Level	Hyper-Parameter	Optimal Value
Network training	Learning rate	0.003245109
Network training	Momentum	0.827834971
KB1 structure	Kernel size	135
	Number of kernels	4
KB2 structure	Kernel size	15
	Number of kernels	12
	Number of blocks	2
KB3 structure	Number of kernels	4

**Table 7:** CNN structure of optimal model for intelligent fault diagnosis.

Layer Number	Layer Type	Activation Size	Number of Learning Parameters
1	Input	12,800 × 1 × 3	0
2	Convolutional	12,800 × 1 × 3	Weigths 135 × 1 × 3 × 4 Bias 1 × 1 × 4
3	Batch normalization	12,800 × 1 × 3	Offset 1 × 1 × 4 Scale 1 × 1 × 4
4	ReLU	12,800 × 1 × 3	0
5	Max. pooling	12,797 × 1 × 4	0
6	Convolutional	12,797 × 1 × 12	Weigths 15 × 1 × 4 × 12 Bias 1 × 1 × 12
7	Batch normalization	12,797 × 1 × 12	Offset 1 × 1 × 12 Scale 1 × 1 × 12
8	ReLU	12,797 × 1 × 12	0
9	Convolutional	12,797 × 1 × 12	Weigths 15 × 1 × 12 × 12 Bias 1 × 1 × 12
10	Batch normalization	12,797 × 1 × 12	Offset 1 × 1 × 12 Scale 1 × 1 × 12
11	ReLU	12,797 × 1 × 12	0
12	Max. pooling	12,794 × 1 × 12	0
13	Convolutional	12,794 × 1 × 12	Weigths 4 × 1 × 12 × 12 Bias 1 × 1 × 12
14	Batch normalization	12,794 × 1 × 12	Offset 1 × 1 × 12 Scale 1 × 1 × 12
15	ReLU	12,794 × 1 × 12	0
16	Max. pooling	12,791 × 1 × 12	0
17	Full-connected	1 × 1 × 16	Weigths 16 × 153,492 Bias 16 × 1
18	Softmax	1 × 1 × 16	0
19	Output	-	0

## Data Availability

The data that support the results of this study are available from the corresponding author, D.K., upon reasonable request.
